# Cumulative evidence of relationships between multiple variants in 8q24 region and cancer incidence

**DOI:** 10.1097/MD.0000000000020716

**Published:** 2020-06-26

**Authors:** Yu Tong, Ying Tang, Shiping Li, Fengyan Zhao, Junjie Ying, Yi Qu, Xiaoyu Niu, Dezhi Mu

**Affiliations:** a Department of Pediatrics; b Key Laboratory of Birth Defects and Related Diseases of Women and Children (Sichuan University), Ministry of Education; c Department of Diagnostic Ultrasound; d Department of Obstetrics and Gynecology, West China Second University Hospital, Sichuan University, Chengdu, Sichuan Province, China.

**Keywords:** 8q24, cancer, genetic variant, meta-analysis, susceptibility

## Abstract

Genome-wide association studies (GWAS) have identified multiple independent cancer susceptibility loci at chromosome 8q24. We aimed to evaluate the associations between variants in the 8q24 region and cancer susceptibility. A comprehensive research synopsis and meta-analysis was performed to evaluate associations between 28 variants in 8q24 and risk of 7 cancers using data from 103 eligible articles totaling 146,932 cancer cases and 219,724 controls. Results: 20 variants were significantly associated with risk of prostate cancer, colorectal cancer, thyroid cancer, breast cancer, bladder cancer, stomach cancer, and glioma, including 1 variant associated with prostate cancer, colorectal cancer, and thyroid cancer. Cumulative epidemiological evidence of an association was graded as strong for DG8S737 -8 allele, rs10090154, rs7000448 in prostate cancer, rs10808556 in colorectal cancer, rs55705857 in gliomas, rs9642880 in bladder cancer, moderate for rs16901979, rs1447295, rs6983267, rs7017300, rs7837688, rs1016343, rs620861, rs10086908 associated in prostate cancer, rs10505477, rs6983267 in colorectal cancer, rs6983267 in thyroid cancer, rs13281615 in breast cancer, and rs1447295 in stomach cancer, weak for rs6983561, rs13254738, rs7008482, rs4242384 in prostate cancer. Data from ENCODE suggested that these variants with strong evidence and other correlated variants might fall within putative functional regions. Our study provides summary evidence that common variants in the 8q24 are associated with risk of multiple cancers in this large-scale research synopsis and meta-analysis. Further studies are needed to explore the mechanisms underlying variants in the 8q24 involved in various human cancers.

## 1 Introduction

The morbidity and mortality of cancers have been increasing worldwide. The genetic factors e.g., a single nucleotide polymorphism have been verified to be associated with the onset of cancers. Identification of genetic factors regulating the development and progression of cancers contributes to improvement of preventive measures and therapeutic outcomes.^[1]^

Genome-wide association studies (GWAS) have identified multiple independent cancer susceptibility loci at chromosome 8q24. These susceptibility loci do not affect coding regions of gene, however, they are in tight LD with many SNPs, often covering large haplotype blocks. The rs6983267, 1 of the variants in 8q24 region was initially identified as a susceptibility locus for colorectal cancer.^[2,3]^ Then multiple loci, such as rs1447295, rs16901979, rs10090154 etc., were confirmed to be associated with prostate cancer.^[4–6]^ In 2008, Eeles et al conducted a two-stage GWAS and identified several alleles associated with prostate cancer on chromosome 8q24.^[7]^ More recently, several breast cancer,^[8,9]^ gliomas,^[10]^ bladder cancer,^[11]^ and stomach cancer^[12]^ risk regions in 8q24 have also been identified. Further study in a large-scale found that rs13281615 G-allele in 8q24 was associated with higher survival rates in breast cancer.^[13]^ In addition, rs9642880 and rs1447295 located in 8q24 region were found to be associated with the risk of bladder^[14]^ and stomach cancer,^[15]^ respectively. In 2014, Skibola et al reported that rs13254990 was associated with follicular lymphoma risk by conducting a large-scale two-stage GWAS.^[16]^

A number of genetic studies have been done to evaluate the contribution of variants in the 8q24 region to risk of human cancer, however, results from these studies were generally inconsistent. In the present study, we performed a comprehensive meta-analysis, involving a total of 146,932 cancer cases and 219,724 controls, to evaluate all genetic studies that investigated associations between variants in the 8q24 region and risk of human cancers.

## 2 Methods

All methods were based on guidelines proposed by the Human Genome Epidemiology Network for systematic review of genetic association studies and followed the guidelines of Preferred Reporting Items for Systematic Reviews and Meta-Analyses.

### 2.1 Search strategy and selection criteria

We systematically searched PubMed and Embase to identify genetic association studies published in print or online before November 30th, 2017 in English language using key terms “8q24” and “variant or polymorphism or genotype” and “cancer or carcinoma or tumor”. The eligibility of each study was assessed independently by 2 investigators (Yu Tong and Ying Tang). The articles included in the meta-analysis must meet the following inclusion criteria:

1. evaluating the associations of genetic variants in the 8q24 with risk of human cancer;
2. providing age-adjusted or multivariate-adjusted risk estimates (e.g., relative risks (RRs), hazard ratios (HRs), odds ratios (ORs), 95% confidence intervals (CIs) or standard errors (SEs)) or sufficient data to calculate these estimates.

Studies were excluded when:

1. they lacked sufficient information;
2. they were not published as full reports, such as conference abstracts and letters to editors;
3. they were studies of cancer mortality (rather than incidence).

### 2.2 Data extraction

Data were extracted by 2 investigators (Yu Tong and Ying Tang), who used recommended guidelines for reporting on meta-analyses of observational studies. Data extracted from each eligible publication included first author, publishing year, study design, method of case selection, source population, ethnicity of participants, sample size, cancer type, variants, major and minor alleles, genotype counts for cases and controls, Hardy-Weinberg equilibrium (HWE) among controls. Ethnicity was classified as African (African descent), Asian (East Asian descent), Caucasian (European descent), or other (including Native Hawaiians, Latinos, Hispanic, etc.) based on the ethnicity of at least 80% of the study population. In total, 103 eligible publications had sufficient data available for extraction and inclusion in meta-analyses. All analyses were based on previous published studies, thus no ethical approval and patient consent are required.

### 2.3 Statistical analysis and assessment of cumulative evidence

The odds ratio was used as the metric of choice for each study. To detect overall genetic associations, allele frequencies were computed for studies reporting allele and genotype data. Pooled odds ratios were computed by the fixed effects model and the random effects model based on heterogeneity estimates. Once an overall gene effect was confirmed, the genetic effects and mode of inheritance were estimated using the genetic model-free approach suggested by Minelli et al. We performed Cochrans Q test and calculated *І*^2^ statistic to evaluate heterogeneity between studies. *І*^2^ values <25% represent no or little heterogeneity, values 25% to 50% represent moderate heterogeneity, and values >50% represent large heterogeneity. Sensitivity analyses were conducted to examine if the significant association would be lost when the first published report was excluded, or studies deviated from HWE in controls were excluded. Harbord test was performed to evaluate publication bias. All analyses were conducted using Stata, version 14.0 (StataCorp, 2017), with the *metan, metabias, metacum*, and *metareg* commands.

Venice criteria^[17]^ was applied to evaluate the epidemiological credibility of significant associations identified by meta-analysis. Credibility was defined in 3 categories: amount of evidence (graded by the sum of test alleles or genotypes among cases and controls: A for >1000, B for 100–1000, and C for <100), replication of the association (graded by the heterogeneity statistic: A for *I*^2^ < 25%, B for *I*^2^ between 25% and 50%, and C for *I*^2^ > 50%), and protection from bias (graded as A: there was no observable bias, and bias was unlikely to explain the presence of the association, B: bias could be present, C: bias was evident or was likely to explain the presence of the association. C was also assigned to an association with a summary OR less than 1.15, unless the association had been replicated by GWAS or GWAS meta-analysis from collaborative studies et al with no evidence of publication bias). Cumulative epidemiological evidence for significant associations was thought to be strong if all 3 grades were A, moderate if all 3 grades were A or B, and weak if any grade was C.

To determine whether a significant association could be excluded as a false positive finding, FPRP (false positive report probability) was calculated by the method described by Wacholder et al FPRP < 0.05, 0.05 ≤ FPRP ≤ 0.20, and FPRP > 0.20 were considered strong, moderate, and weak evidence of true association, respectively.

### 2.4 Functional annotation

We conducted analyses to evaluate the potential functional effect of variants on 8q24 using data from the Encyclopedia of DNA Elements (ENCODE) Project and performed functional annotation for variants significantly associated with cancer risk through the UCSC Genome browser (http://genome.ucsc.edu/).

## 3 Results

### 3.1 Characteristics of the studies included in this meta-analysis

**Figure 1:**
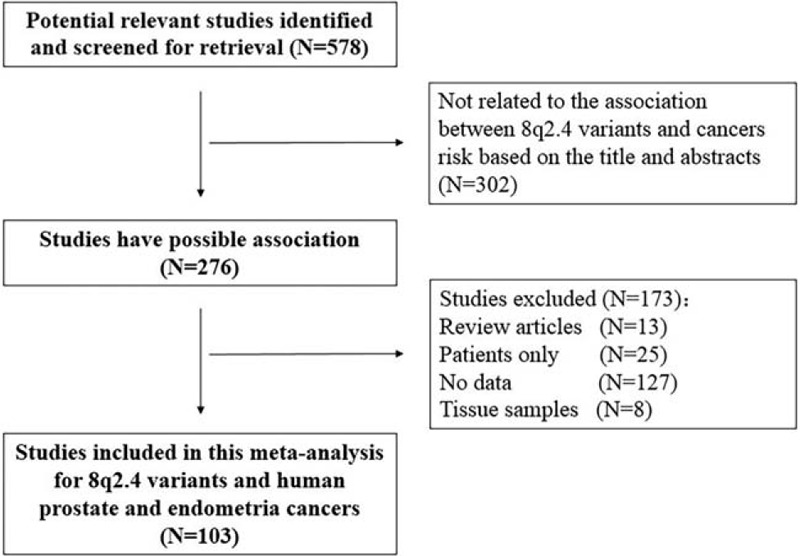
Flow diagram of included and excluded studies.

Our search yielded a total of 578 publications. Based on a review of titles and abstracts, 276 articles were retained. The full text of these 276 articles were reviewed in detail, and 103 studies were eligible for inclusion in the meta-analysis. The specific process for identifying eligible studies and inclusion and exclusion criteria are summarized in Figure 1. Characteristics of the included articles were presented in Table 1.   

**Table 1:**
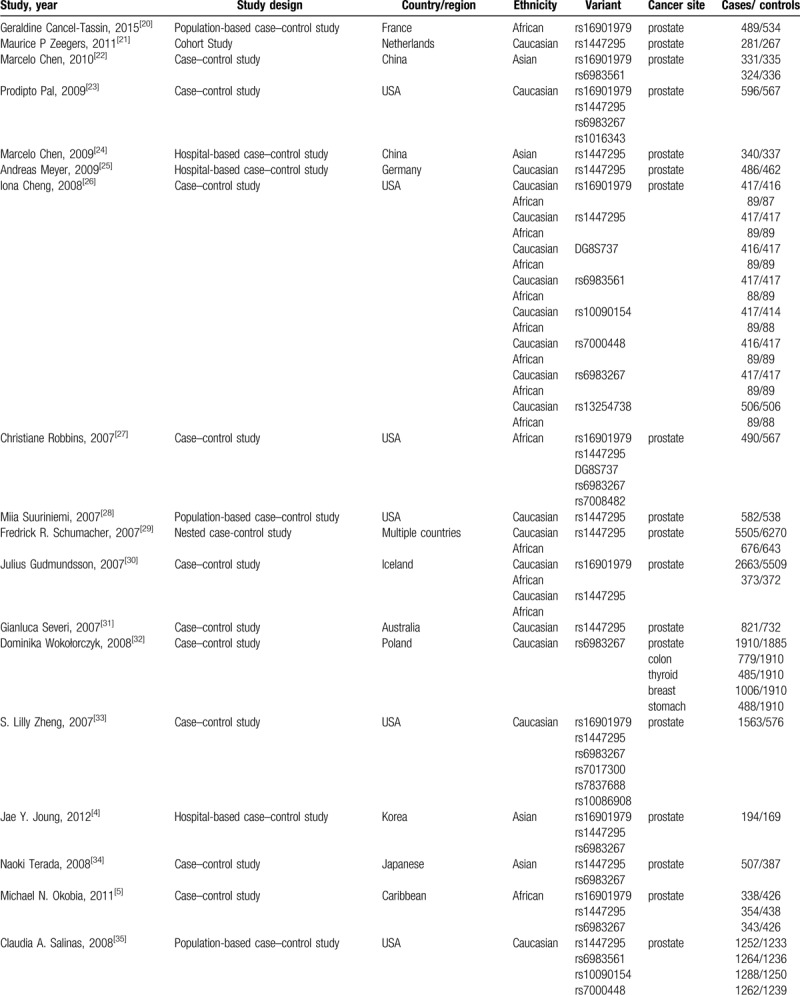
Characteristics of the included articles.

**Table 1 (Continued):**
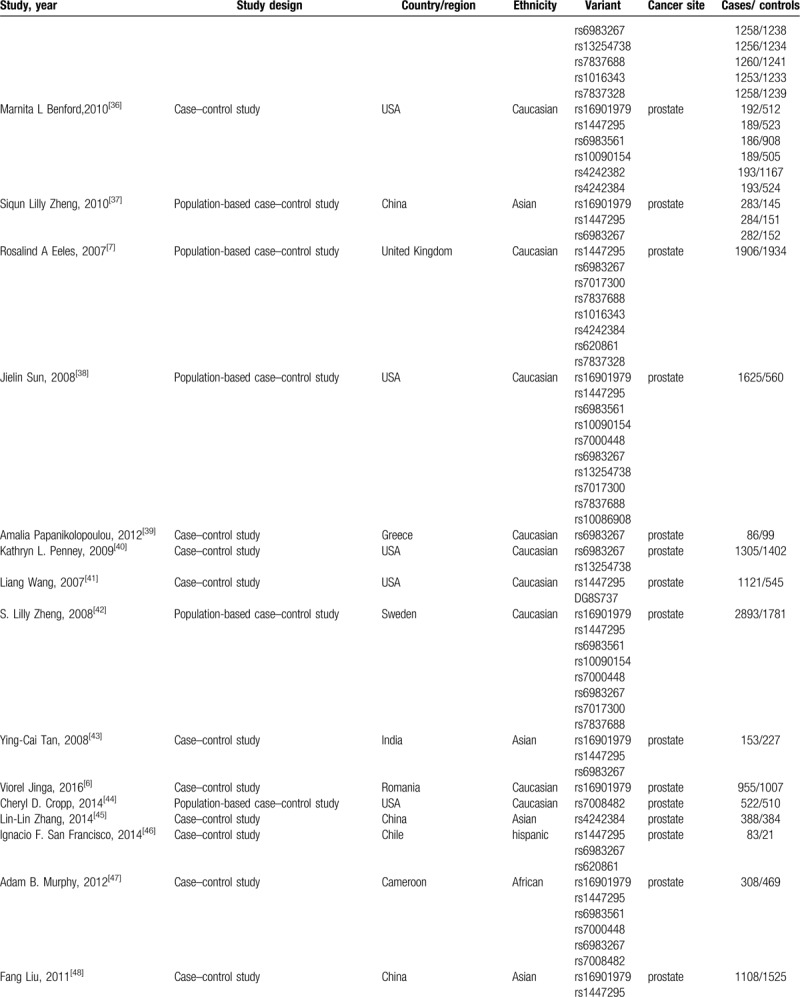
Characteristics of the included articles.

**Table 1 (Continued):**
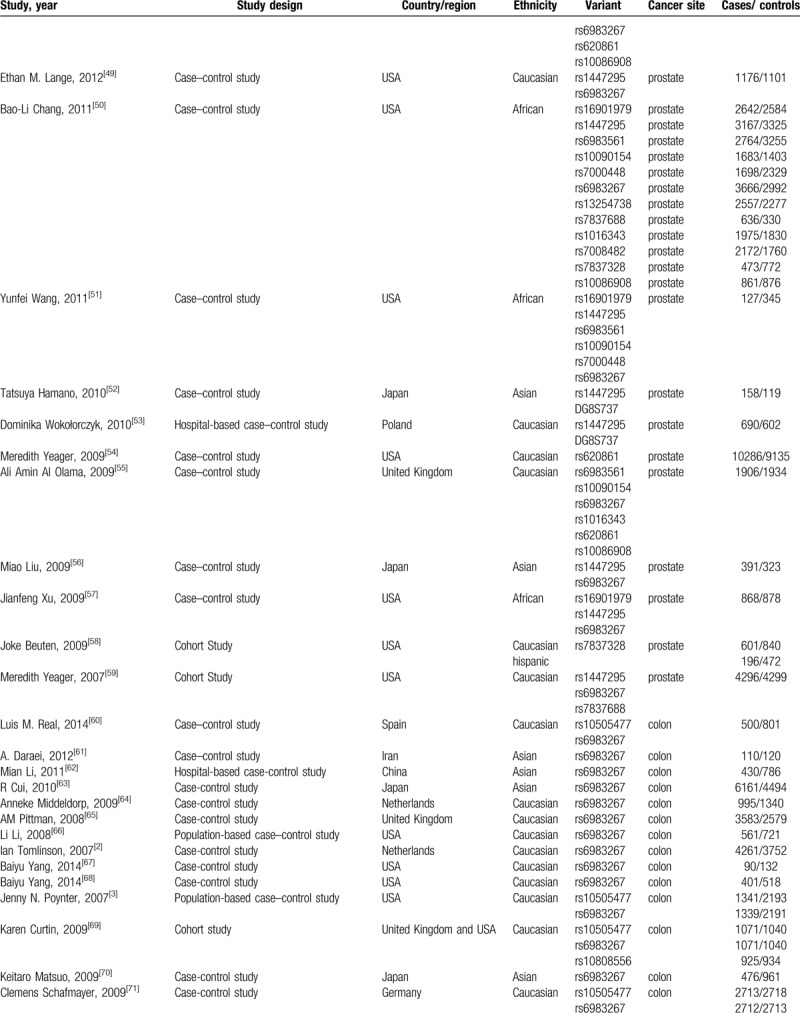
Characteristics of the included articles.

**Table 1 (Continued):**
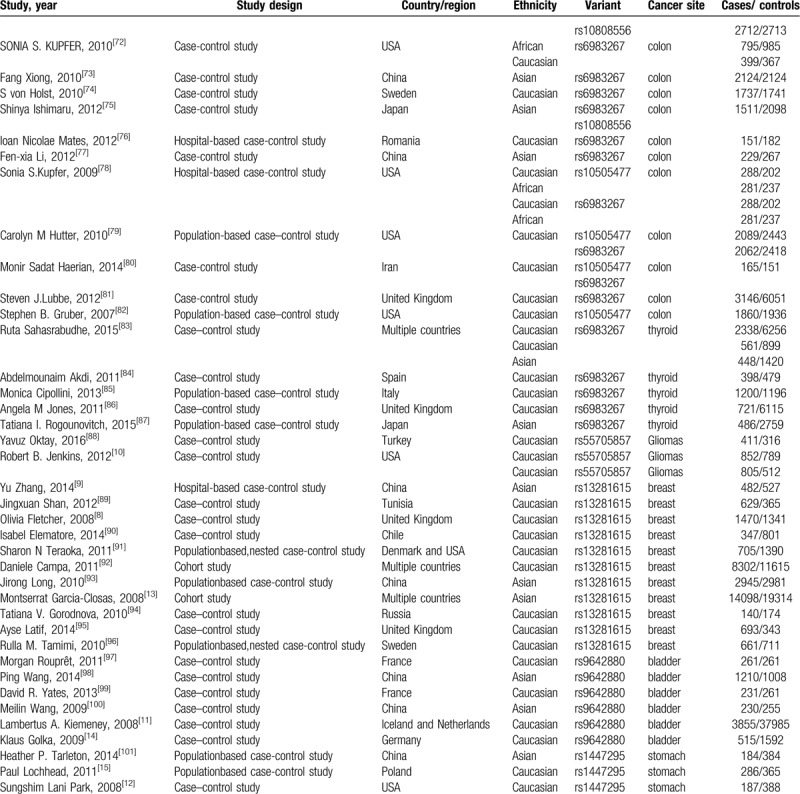
Characteristics of the included articles.

### 3.2 Associations between 8q24 variants and cancer risk

A summary of the meta-analysis findings regarding associations between 8q24 variants and cancer risk was shown in Table 2. Totally, 20 variants were nominally significantly associated with risk of prostate cancer, colorectal cancer, thyroid cancer, breast cancer, bladder cancer, stomach cancer, and gliomas (*P* < .05). Significant associations with prostate cancer risk were found for rs16901979 (OR = 1.456, 95% CI: 1.31–1.64; *P* = 1.12 × 10^–11^), rs1447295 (OR = 1.29, 95% CI: 1.21–1.38; *P* = 2.74 × 10^–14^), DG8S737 -8 allele (OR = 1.29, 95% CI: 1.12–1.48; *P* = 2.83 × 10^–4^), rs6983561 (OR = 1.29, 95% CI: 1.02–1.64; *P* = .04), rs10090154 (OR = 1.33, 95% CI: 1.17–1.52; *P* = 1.87 × 10^–5^), rs7000448 (OR = 1.11, 95% CI: 1.04–1.19; *P* = .003), rs13254738 (OR = 1.11, 95% CI: 1.01–1.22; *P* = .026), rs6983267 (OR = 1.14, 95% CI: 1.04–1.25; *P* = .006), rs7017300 (OR = 1.39, 95% CI: 1.15–1.68; *P* = .001), rs7837688 (OR = 1.48, 95% CI: 1.29–1.71; *P* = 4.76 × 10^–8^), rs1016343 (OR = 1.33, 95% CI: 1.20–1.48; *P* = 5.64 × 10^–8^), rs7008482 (OR = 0.77, 95% CI: 0.62–0.96; *P* = .021), rs4242384 (OR = 1.42, 95% CI: 1.05–1.92; *P* = .022), rs620861 (OR = 0.84, 95% CI: 0.77–0.92; *P* = 7.49 × 10^–5^), rs10086908 (OR = 0.73, 95% CI: 0.60–0.88; *P* = .001). Significant associations with colorectal cancer risk were found for rs10505477 (OR = 1.13, 95% CI:1.09–1.18; *P* = 7.03 × 10^–11^), rs6983267 (OR = 1.17, 95% CI:1.08–1.19; *P* = 4.66 × 10^–7^) and rs10808556 (OR = 1.18, 95% CI:1.12–1.25; *P* = 2.10 × 10^–9^).

**Table 2:**
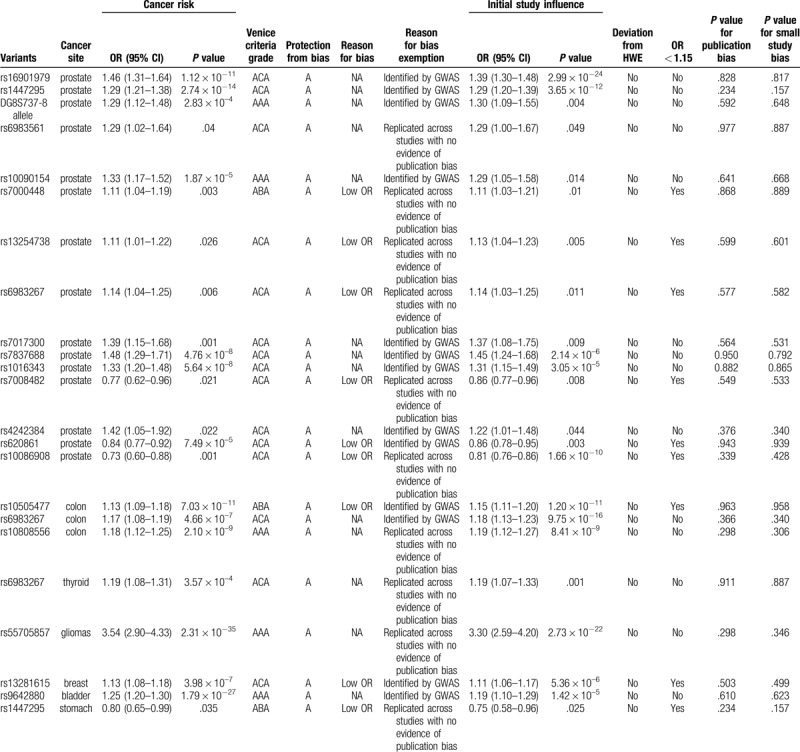
Details of protection from bias for genetic variants significantly associated with cancer risk in meta-analyses.

Significant associations with thyroid cancer risk were found for rs6983267 (OR = 1.19, 95% CI: 1.08–1.31; *P* = 3.57 × 10^–4^). Significant associations with gliomas risk were found for rs55705857 (OR = 3.54, 95% CI: 2.90–4.33; *P* = 2.31 × 10^–35^). Significant associations with breast cancer risk were found for rs13281615 (OR = 1.13, 95% CI: 1.08–1.18; *P* = 3.98 × 10^–7^). Significant associations with bladder cancer risk were found for rs9642880 (OR = 1.25, 95% CI: 1.20–1.30; *P* = 1.79 × 10^–27^). Significant associations with stomach cancer risk were found for rs1447295 (OR = 0.80, 95% CI: 0.65–0.99; *P* = .035). No significant associations were found for rs4242382, rs4645959, rs7837328, rs16901966, rs10505476, rs13281615 with prostate cancer risk, rs1447295, rs7837328, rs10090154 with colorectal cancer risk, rs4295627 with gliomas risk, rs1562430, rs6983267 with breast cancer risk and rs6983267 with stomach cancer risk (data not shown).

### 3.3 Heterogeneity, sensitivity analysis and bias

As shown in Table 2, no or little heterogeneity was observed for associations of DG8S737 -8 allele (*I*^2^ = 2.32%, *P* = .803) and rs10090154 (*I*^2^ = 0.0%, *P* = .873) with prostate cancer, rs10808556 (*I*^2^ = 0.0%, *P* = .394) with colorectal cancer, rs55705857 (*I*^2^ = 10.9%, *P* = .326) for gliomas, rs9642880 (*I*^2^ = 4.10%, *P* = .39) with bladder cancer.

Moderate heterogeneity was observed for associations of rs7000448 (*I*^2^ = 36.2%, *P* = .152) with prostate cancer, rs10505477 (*I*^2^ = 29.2%, *P* = .185) with colorectal cancer and rs1447295 (*I*^2^ = 28.0%, *P* = .249) with stomach cancer.

Large heterogeneity was found for associations of rs16901979 (*I*^2^ = 84.3%, *P* = .000), rs1447295 (*I*^2^ = 77.6%, *P* = .000), rs6983561 (*I*^2^ = 92.2%, *P* = .000), rs6983267 (*I*^2^ = 90.5%, *P* = .000), rs13254738 (*I*^2^ = 59.8%, *P* = .029), rs7017300 (*I*^2^ = 83.3%, *P* = .000), rs7837688 (*I*^2^ = 80.1%, *P* = .000), rs1016343 (*I*^2^ = 70.2%, *P* = .009), rs7008482 (*I*^2^ = 69.2%, *P* = .039), rs4242384 (*I*^2^ = 81.3%, *P* = .005), rs620861 (*I*^2^ = 73.1%, *P* = .005), rs10086908 (*I*^2^ = 89.3%, *P* = .000) with prostate cancer, rs6983267 with colorectal cancer (*I*^2^ = 64.4%, *P* = .000) and rs6983267 with thyroid cancer (*I*^2^ = 78.6%, *P* = .000), rs13281615 (*I*^2^ = 58.9%, *P* = .007) with breast cancer.

We also performed sensitivity analysis to evaluate the stability of results of these associations and found that removal of a single study, the first published or studies deviated from HWE in controls did not change the summary ORs (Table 2).

### 3.4 Cumulative evidence of association

**Figure 2:**
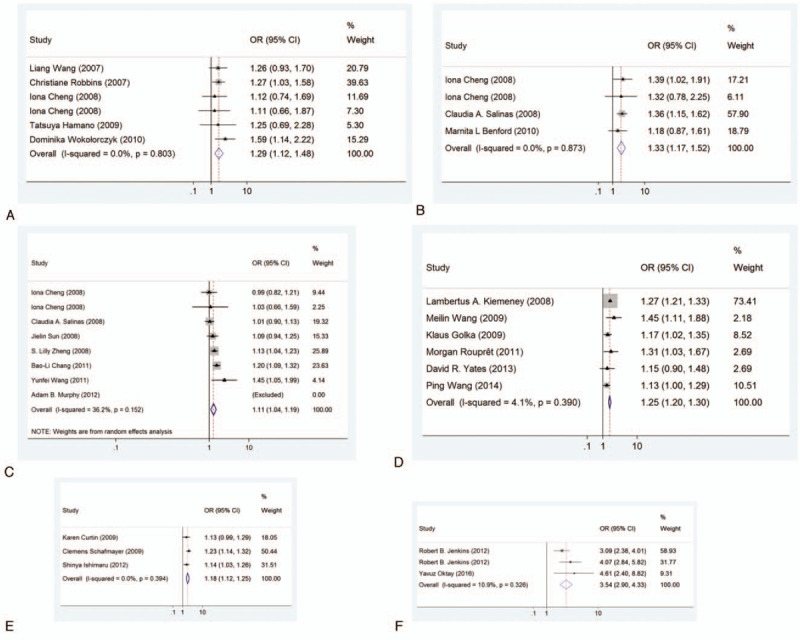
Forest plots for associations between selected variants in the 8q24 region and cancer risk. A: Associations of DG8S737-8 allele with prostate cancer risk. B: Associations of rs10090154 with prostate cancer risk. C: Associations of rs7000448 with prostate cancer risk. D: Associations of rs9642880 with bladder cancer risk. E: Associations of rs10808556 with colorectal cancer risk. F: Associations of rs55705857 with gliomas. The OR of each study is represented by a square, and the size of the square represents the weight of each study with respect to the overall estimate. 95% CIs are represented by the horizontal lines, and the diamond represents the overall estimate and its 95% CI.

Epidemiological credibility was graded for the 23 identified significant associations (Table 2). Venice criteria was first applied to evaluate these associations. Strong for evidence of true association with cancer risk were assigned to DG8S737 -8 allele, rs10090154 in prostate cancer, rs10808556 in colorectal cancer, rs55705857 in gliomas, rs9642880 in bladder cancer, moderate were assigned to rs7000448 in prostate cancer, rs1447295 in stomach cancer, weak were assigned to other variants. We next evaluated the probability of true association with cancer risk for the nominally significant variants through calculating the FPRP value. Associations with cancer risk had a FPRP value < 0.05 for 18 variants (rs16901979, rs1447295, DG8S737 -8 allele, rs10090154, rs7000448, rs6983267, rs7017300, rs7837688, rs1016343, rs620861, rs10086908 in prostate cancer, rs10505477, rs6983267, rs10808556 in colorectal cancer, rs6983267 in thyroid cancer, rs55705857 in gliomas, rs13281615 in breast cancer, rs9642880 in bladder cancer), FPRP value 0.05 - 0.20 for 3 variants (rs13254738, rs4242384 in prostate cancer, rs1447295 in stomach cancer), and FPRP value >0.20 for rs6983561, rs7008482 in prostate cancer. Based on the FPRP value, we upgraded cumulative evidence from moderate to strong for rs7000448 in prostate cancer, weak to moderate for rs16901979, rs1447295, rs6983267, rs7017300, rs7837688, rs1016343, rs620861, rs10086908 associated with prostate cancer, rs10505477, rs6983267 with colorectal cancer, rs6983267 with thyroid cancer, and rs13281615 with breast cancer. Altogether, cumulative epidemiological evidence of an association was graded as strong for DG8S737 -8 allele (Fig. 2A), rs10090154 (Fig. 2B), rs7000448 (Fig. 2C) in prostate cancer, rs9642880 in bladder cancer (Fig. 2D), rs10808556 in colorectal cancer (Fig. 2E), rs55705857 in gliomas (Fig. 2F), moderate for rs16901979, rs1447295, rs6983267, rs7017300, rs7837688, rs1016343, rs620861, rs10086908 associated in prostate cancer, rs10505477, rs6983267 in colorectal cancer, rs6983267 in thyroid cancer, rs13281615 in breast cancer, and rs1447295 in stomach cancer, weak for rs6983561, rs13254738, rs7008482, rs4242384 in prostate cancer.

### 3.5 Functional annotation

**Figure 3:**
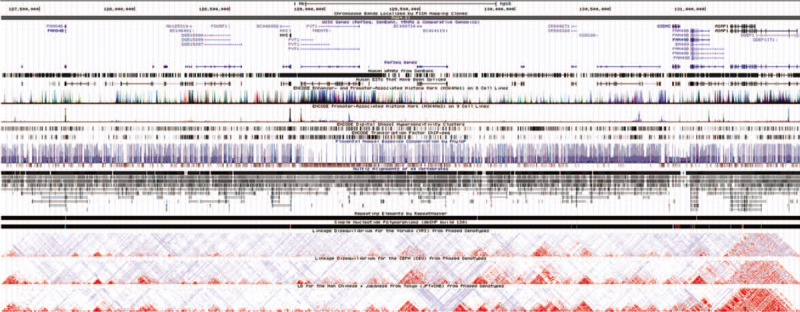
Evidence from ENCODE data for regulatory function of SNPs in 8q24 using the UCSC Genome Browser. The plot represent 8q24.21 region (NCBI Human Genome GRCh37). Tracks (from top to bottom) in each of the plots are Genome Base Position, Chromosome Bands, UCSC Genes, Human mRNAs from GenBank, Human ESTs That Have Been Spliced, ENCODE Enhancer- and Promoter-Associated Histone Mark (H3K4Me1) on 8 Cell Lines, ENCODE Promoter-Associated Histone Mark (H3K4Me3) on 9 Cell Lines, ENCODE Digital DNaseI Hypersensitivity Clusters, ENCODE Transcription Factor ChIP-seq, ENCODE Chromatin State Segmentation by HMM from Broad Institute, Simple Nucleotide Polymorphisms (dbSNP build 130), Linkage Disequilibrium (LD) for the Yoruba (YRI) from Phased Genotypes, LD for the CEPH (CEU) from Phased Genotypes, and LD for the Han Chinese+Japanese from Tokyo (CHB+JPT) from Phased genotypes.

Data from the ENCODE Project suggested that variants located at 8q24 might be located in a region with strong enhancer activity and DNase I hypersensitivity site. The LD plots indicated that the genetic structure of and African ancestry (Fig. 3).

## 4 Discussion

To our knowledge, this study is the largest and most comprehensive assessment of literatures on associations between genetic variants in the 8q24 region and cancer risk. Preliminary meta-analyses were mostly limited to a single SNP in relation to 1 cancer. Here we performed a research synopsis and meta-analysis to systematically evaluate associations between variants in 8q24 region and risk of 7 human cancers using data from 103 articles total 146,932 cancer cases and 219,724 controls. Our study not only provides an update of the variants analyzed previously, but also evaluates more variants that have not been analyzed in previous meta-analyses.

Of the 28 variants located in 8q24, 20 were significantly associated with risk of prostate cancer, colorectal cancer, thyroid cancer, breast cancer, bladder cancer, stomach cancer and glioma, including 1 variant associated with prostate cancer, colorectal cancer and thyroid cancer. Using the Venice criteria and false-positive report probability tests, we graded 6 variants (DG8S737 -8 allele, rs10090154, rs7000448 in prostate cancer, rs10808556 in colorectal cancer, rs55705857 in gliomas, rs9642880 in bladder cancer) strong for cumulative evidence of significant associations with cancer risk. In addition, we performed functional annotation for variants significantly associated with cancer risk using data from the ENCODE Project and the UCSC Genome browser and found that these variants might be located in a region with strong enhancer activity and DNase I hypersensitivity site.

Multiple genetic variants on chromosome 8q24 have been reported to be significantly associated with an increased susceptibility to prostate, colorectal, breast cancer, et al. These risk loci are located in a cancer-associated regions “gene desert”, a few hundred kilobases telomeric to the Myc gene. It was predicted that these risk-associated variants could affect the regulation or transcription of the gene, such as MYC, TCF7L2, FAM84B, et al outside the 8q24 region. Another speculation is that some risk-associated variants are linked to these risk-associated SNPs. In 2010, Sotelo et al found that there are enhancer elements located within the cancer-associated regions can regulate Myc promoter activity, and the previously identified cancer risk locus, rs6983267, located within this enhancer, acts as a functional variant in the regulation of Myc transcription.^[18]^ Soon after, Hazelett and his colleagues reported that the G allele at rs183373024 may result in the downregulation of a tumorsuppressor-like gene target of the FoxA1 enhancer.^[19]^ Therefore, 8q24 can be viewed as an enhancers region affecting cancer risk via the regulation of distant gene expression. Our study revealed strong evidence of an association with cancer risk for 6 variants, indicating that there might be different causal variants and functional mechanisms involved in associations of variants in the 8q24 with risk of human cancers.

There are several limitations of the study. First, a unified analysis standard across studies such as the control, could not be defined for lack of raw data from the original publications. Second, it is likely that some publications were overlooked, some relevant published studies with null results may not be identified. Third, due to insufficient data, we were unable to evaluate publication bias for associations between several variants in 8q24 region and cancer. Finally, we conducted meta-analysis based on minor allele of a variant, future studies with much larger sample size are warranted to confirm these associations.

## 5 Conclusions

Our study provides summary evidence that common variants in the 8q24 are associated with risk of prostate cancer, colorectal cancer, thyroid cancer, breast cancer, bladder cancer, stomach cancer, and glioma in this large-scale research synopsis and meta-analysis, suggesting that variants in the 8q24 region are related mechanistically to the development of cancer. Interactions of SNP-SNP, gene–gene, and gene–environment should be addressed in future large multicentric studies to explore the mechanisms underlying variants in the 8q24 involved in various human cancers.

## Author contributions

**Data curation:** Yu Tong, Ying Tang, Junjie Ying.

**Software:** Shiping Li, Fengyan Zhao, Yi Qu.

**Writing – original draft:** Yu Tong.

**Writing – review & editing:** Dezhi Mu, Xiaoyu Niu.
